# 

**Published:** 1912-02-17

**Authors:** 


### Appointments.

Mr. E. P. Hudson, L.D.S., has been appointed Dental Surgeon to the Royal Free Hospital.

Mr. E. H. Jones, M.B., B.S. Lond., M.R.C.S. Eng., L.R.C.P. Lond., has been appointed House Surgeon to the Northampton General Hospital.

Mr. Fredric Battinson Smith, M.R.C.S., L.R.C.P., has been appointed house physician to the Warneford, Leamington, and South Warwickshire General Hospital, Leamington Spa.

Mr. Reginald Pollard, M.B., M.R.C.S., D.P.H., has been elected an anæsthetist to the West-End Hospital, Welbeck Street Mr. Pollard also holds the post of anæsthetist to the Royal National Orthopædic Hospital, and has been a clinical assistant to the West-End Hospital for about a year.

The following house appointments have been made at St. Thomas's Hospital from February 20, subject to the approval of the Treasurer: To be Casualty Officers and Resident Anæsthetists: Mr. C. V. Anderson, M.R.C.S., L.R.C.P.; Mr. H. C. Bazett, B.A., M.B., B.Ch. Oxon., F.R.C.S. Eng.; Mr. J. L. Birley, B.A. Oxon., M.R.C.S., L.R.C.P.; Mr. A. D. Gardner, M.B., B.Ch. Oxon., M.R.C.S., L.R.C.P.; Mr. N. F. Lock, B.A. Cantab., M.R.C.S., L.R.C.P.; and Mr. W. B. Laird, M.R.C.S., L.R.C.P. To be House Surgeon to Block 8: Mr. A. K. Hamilton, M.R.C.S., L.R.C.P. To be Obstetric House Physicians: (Sen.), Mr. E. M. Lauderdale, B.A. Cantab., M.R.C.S., L.R.C.P.; and (Jun.) Mr. J. F. Taylor, B.A. Cantab., M.R.C.S., L.R.C.P. To be Ophthalmic House Surgeon: (Sen.) Mr. F. R. B. Skrimshire, M.R.C.S., L.R.C.P.

### BOOKS RECEIVED.

Baillière, Tindall and Cox: "Gynæcological Nursing," by A. E. Giles, M.D.; "Wellcome Tropical Research Laboratories. Fourth Report," Balfour; "Lectures on Midwifery for Midwives," by A. B. Calder.

J. and A. Churchill: "Poliomyelitis in Relation to the Spread of Infection by Schools," by F. E. Batten; "The Tobacco Habit," by H. H. Tidsdall.

Chatto and Windus: "London Charities," by Herbert Fry.

A. C. Black: "Who's Who, 1912": "The Writers' and Artists' Year Book, 1912": "The Englishwoman's Year Book"; "Who's Who Year Book, 1912-13."

W. B. Saunders Company: "Reference Handbook of Obstetric Nursing," by W. Reynolds Wilson; "The Pocket Medical Dictionary," by W. A. Newman Dorland.

### OUR BUREAU OF INFORMATION.

### Answers to Correspondents.

### COMPENSATION AND NATIONAL INSURANCE.

"WORKING MAN" writes: Is it true that if I am injured I cannot get compensation under the Workmen's Compensation Act if I am under the National Insurance Act?

(This statement is not quite accurate. If a man injured in the course of his employment is under the Workmen's Compensation Act awarded compensation amounting to ten shillings a week or more, he will receive nothing under the Insurance Act; but if the compensation is less than ten shillings a week he will, under the Insurance Act, receive enough to bring it up to that amount.)

### Rules for Correspondents.

Everyone who uses or wishes to help our Bureau of Information must be kind enough to observe the following rules:—

(1) Postal replies cannot be given. Exception will only be made in very special cases at the Editor's discretion.

(2) Queries or answers relating to different cases must be written, or preferably typed, on separate sheets of paper, and the writing must never be crossed.

(3) Questions and replies received not later than Wednesday will, as far as possible, be dealt with in our issue of the following Saturday week.

(4) Letters must have attached the name and address of the sender, with a pseudonym for publication if preferred.

(5) Applications referring to non-dependents where the need relates to business or employment must be accompanied by a postal order for 5s., when the requirement shall have publicity in these columns.

(6) All applicants for insertion in our list of Approved Homes must send a registration fee of five shillings. The omission of this leads to delay and gives avoidable trouble.

(7) *All communications* for this department *must be accompanied by the coupon* to be found at the bottom of the third page of the cover on the inside, and should be addressed to the Editor of the Bureau of Information, c/o THE HOSPITAL, 29 Southampton Street, Strand, London, W.C.

### The Week's Novelties.

SHIPS' SURGEONS AND DOCTORS' HOLIDAYS.

THE Peninsular and Oriental Company announces fifteen cruises for the current year: nine by the *Vectis*, beginning with a departure from Southampton on the 21st inst., for Lisbon, Algiers, Athens, Palestine, Egypt, and Naples, and six by their cruising steamer *Mantua* (11,500 tons), beginning with a departure from London in May for the Azores, Madeira, Canary Islands, etc. In connection with these cruises the company has issued an illustrated programme giving details of the various itineraries and fares, which may be obtained from their offices.

ELECTRIC STERILISERS.

THE A.E.G. Electric Company, known to the hospital world as manufacturers of all electric appliances for hospital and institutional use, are now inviting attention to their electric sterilisers. In modern theatres where electricity is the illuminant, and perhaps even supplies the heating, the advantages of all sources of energy for all purposes in the theatre unit is obvious. In the future electricity almost certainly will be used for all cookery, and to-day, where electricity is already installed, expense being no immediate object in the theatre, electric sterilisers advertise their own claim. The address to write to for full particulars is 133-135 Oxford Street, W. An illustration may be seen among our advertisements this week.

### National Health Insurance Commission.

THE Insurance Commissioners are issuing to Registered Friendly Societies, Trade Unions, etc., Circular No. A.S. 1 with regard to the approval of societies. Copies of the circular are being sent to all those societies which appear on the lists available, but the Insurance Commissioners are aware that there are many societies (including unregistered societies and bodies which may contemplate forming a new society for the purposes of the National Health Insurance Act) whose names do not appear on any list.

Societies which do not receive within a few days copies of the circular are requested to consider this publication as a notice on which they can act. If they desire copies of the circular and of the forms referred to in it, they should write to the National Health Insurance Commissions in London, Edinburgh, Dublin, or Cardiff, as the case may be.

### Florence Nightingale Memorial.

WE have to acknowledge the receipt of the following contributions:—Wm. Koch, Esq. (annuities), £10 10s.; City of Bradford Co-operative Society, £2 2s.; Sister Mary Cameron (annuities), 14s. Received by Mr. G. Q. Roberts: Per Sir Henry Burdett's private collection and appeal in THE HOSPITAL (£43 15s. of this earmarked annuities), £766 17s. 10d.; The Rifle Brigade Club, £20; Sir Edwin Durning Lawrence, Bart. (annuities), £10 10s.; Mrs. Shore Nightingale, £10 10s.; Mary Countess of Lovelace (statue), £10; Mr. and Mrs. Williams, £10; Lionel Robinson, Esq., £5; The Hon. Mrs. Walrond (annuities), £5; Mrs. George Cornwallis-West, £5; George J. Drummond, Esq., £5; Miss S. F. Ramsden, £5; T. Arnold Herbert, Esq., £5; Sir W. H. Hornby, Bart., £5; the Lord Huntingfield, £5; Miss E. G. Mackie, £5; Lady Stephenson, £3 3s.; Mrs. Mounsey-Heysham (statue), £3; Mrs. Pearson, £3; Edgar Horne, Esq., £2 2s.; General Sir George Higginson, G.C.B., £2 2s.; Susan Countess of Southesk (annuities), £2 2s.; Mrs. Whitley, £2 2s.; Sir Gerard Lowther, K.C.M.G., £2 2s.; Rear-Admiral Sir George Warrender, £2; General Sir A. H. W. Williams, £2; the Viscount Dillon, £2; Miss Campbell's collection, £2 2s. 6d.; Lady Turner, £2 2s.; Lieutenant-General Sir James Hills-Johnes (statue), £1 1s.; Lady Hills-Johnes (statue), £1 1s.; Mrs. Warner, £1 1s.; A. J. Webbe, Esq., £1 1s.; Sir George Hingley, Bart., £1 1s.; Rev. Canon Egerton Leigh, £1 1s.; the Lady Morrish, £1 1s.; Colonel H. B. Mortimer, £1 1s.; A. C. Ainger, Esq., £1 1s.; Mrs. F. J. Burton, £1 1s.; Sir Reginald Graham, Bart., £1 1s.; Dr. C. H. Herford, £1 1s.; R. S. Gundry, Esq., C.B., £1 1s.; Colonel Hon. William Lambton, £1 1s.; Miss C. M. Wigram, £1 1s.; F. S. Wigram, Esq., £1 1s.; Mrs. Sykes, £1 1s.; Lieutenant-Colonel Edward Davis, R.A.M.C., £1 1s.; P. I. D. Wykeham, Esq., £1; Miss Brocklehurst, £1; C. S. Loch, Esq., £1; Mrs. Lane (statue), £1; Colonel John Hinde, C.B. (statue), 10s. 6d.; Mrs. Turner, 10s. 6d.; Staff of the Salisbury Training College for Mistresses, 10s.; The Cottage Hospital, Fleet, Hants, 7s.; Mrs. George Beauchamp's Collection, 7s.; Staff of Military Hospital, Bodmin, 6s.; John R. Surtees, Esq., 5s.; J. R. Swinglehurst, Esq. (annuities), 5s.; Miss E. Elder, 5s.; C. H. Rowe, Esq., 5s.; Miss Penelope Hastings, 5s.; Staff of Military Hospital, Hilsea, 3s. 6d.; Nurse Elizabeth Arnold (annuities), 2s. 6d.; Miss Florence E. Minns, 2s. 6d.; The *Times* Book Co., Ltd. (from sale of Florence Nightingale booklets), 2s.; Rev. Canon G. E. Hasell (annuities), 1s.; Nurse E. E. Hughes, 1s.

